# 2,2′-Dimethyl-4,4′-(sulfonyldi-*p*-phenyl­ene)dibut-3-yn-2-ol dihydrate

**DOI:** 10.1107/S160053680804350X

**Published:** 2009-01-08

**Authors:** Shi-xu Yi, Jian Men, Yang Wang, Yong Xiao, Guo-wei Gao

**Affiliations:** a College of Chemistry, Sichuan University, Chengdu 610064, People’s Republic of China

## Abstract

The asymmetric unit of the title compound, C_22_H_22_O_4_S·2H_2_O, contains one quarter of the organic mol­ecule and one half water mol­ecule, the site symmetries of the S atom and the water O atom being *mm2* and *m*, respectively. The dihedral angle between the benzene rings is 76.27 (11)°. In the crystal structure, inter­molecular O—H⋯O hydrogen bonds link the mol­ecules into chains running parallel to the *a* axis.

## Related literature

For the properties and synthesis of thermosetting acetyl­ene-terminated resins, see: Lu & Hamerton (2002 ▶). For the applications of the title compound, see: Hanson & Millburn (1984 ▶); Poon *et al.* (2006 ▶).
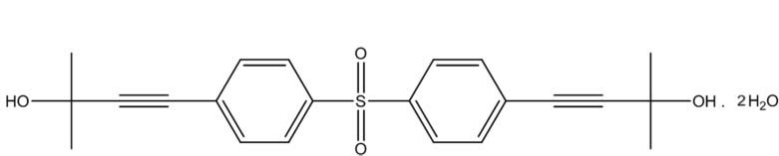


## Experimental

### 

#### Crystal data

C_22_H_22_O_4_S·2H_2_O
*M* *_r_* = 418.50
Orthorhombic, 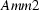

*a* = 19.751 (3) Å
*b* = 10.904 (3) Å
*c* = 5.092 (2) Å
*V* = 1096.5 (7) Å^3^
*Z* = 2
Mo *K*α radiation
μ = 0.18 mm^−1^
*T* = 292 (2) K
0.46 × 0.20 × 0.16 mm

#### Data collection

Enraf–Nonius CAD-4 diffractometer
Absorption correction: spherical (*WinGX*; Farrugia, 1999 ▶) *T*_min_ = 0.921, *T*_max_ = 0.972
1211 measured reflections
888 independent reflections
756 reflections with *I* > 2σ(*I*)
*R*_int_ = 0.014
3 standard reflections every 50 reflections intensity decay: 1.2%

#### Refinement

*R*[*F* ^2^ > 2σ(*F* ^2^)] = 0.053
*wR*(*F* ^2^) = 0.142
*S* = 1.09
888 reflections
81 parameters
3 restraints
H atoms treated by a mixture of independent and constrained refinement
Δρ_max_ = 0.52 e Å^−3^
Δρ_min_ = −0.40 e Å^−3^
Absolute structure: Flack (1983 ▶); 284 Friedel pairs
Flack parameter: −0.1 (2)

### 

Data collection: *DIFRAC* (Gabe & White, 1993 ▶); cell refinement: *DIFRAC*; data reduction: *NRCVAX* (Gabe *et al.*, 1989 ▶); program(s) used to solve structure: *SHELXS97* (Sheldrick, 2008 ▶); program(s) used to refine structure: *SHELXL97* (Sheldrick, 2008 ▶); molecular graphics: *ORTEP-3 for Windows* (Farrugia, 1997 ▶); software used to prepare material for publication: *SHELXL97*.

## Supplementary Material

Crystal structure: contains datablocks global, I. DOI: 10.1107/S160053680804350X/rz2282sup1.cif

Structure factors: contains datablocks I. DOI: 10.1107/S160053680804350X/rz2282Isup2.hkl

Additional supplementary materials:  crystallographic information; 3D view; checkCIF report

## Figures and Tables

**Table 1:** Hydrogen-bond geometry (Å, °)

*D*—H⋯*A*	*D*—H	H⋯*A*	*D*⋯*A*	*D*—H⋯*A*
O3*W*—H1*W*⋯O2^i^	0.78	2.07	2.800 (6)	157

Symmetry code: (i) 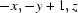.

**Fig. 1.**
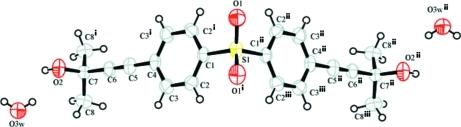
The molecular structure of the title compound, with displacement ellipsoids drawn at the 30% probability level [symmetry codes: (i) = x, 1-y, z; (ii) = 1-x, 1-y, z; (iii) = 1-x, y, z].

## supplementary crystallographic information

### Comment

Acetylene-terminated resins are commercially employed in composite materials, in particular where high strength, light weight materials capable of withstanding high temperatures are required (Lu & Hamerton, 2002). The title compound is an important intermediate for the preparation of these acetylene-terminated compounds (Hanson & Millburn, 1984) and a series of luminescent and thermally stable materials (Poon *et al.*, 2006). We report here the synthesis and crystal structure of the title compound (Fig. 1).

The asymmetric unit of the title compound contains one fourth of the organic molecule and one half water molecule, the site symmetries of the S1 sulphur atom and the O3W water oxygen atom being *mm2* and *m*, respectively. The dihedral angles between the benzene rings is 103.73 (11)°. The displacement of the C7 atom of the 2-hydroxy-2-methyl-4-but-3-ynyl substituent from the plane of the aromatic ring is -0.1870 (14) Å. In the crystal structure, the water molecules and the hydroxy groups are involved in the formation of intermolecular O—H···O hydrogen bonds (Table 1) forming chains running parallel to the *a* axis.

### Experimental

1,1'-Sulfonylbis(4-iodobenzene) (10.00 g, 21.28 mmol), triethylamine (100 ml), PdCl_2_(PPh_3_)_2_ (0.02 g, 0.03 mmol), PPh_3_ (0.04 g, 0.15 mmol), 2-methylbut-3-yn-2-ol (4.29 g, 51.10 mmol) and CuI (0.04 g, 0.21 mmol) were added to a 250 ml three-ecked flask, and the mixture heated to reflux for 10 h. After completion of the reaction, the mixture was filtered and the filtrate was evaporated under reduced pressure. Single crystals suitable for X-ray analysis were obtained by slow evaporation of an ethanol/ water solution (10:1 v/v) (6.90 g, 85% yield; m.p. 435–437 K).

### Refinement

The hydroxy H atom was located in a difference Fourier map and refined isotropically with the O—H distance restrained to 0.82Å. The water H atom was located in a difference Fourier map and refined as riding. All other H atoms were positioned geometrically and refined using a riding model, with C—H = 0.93–0.96 Å and *U*_iso_(H) = 1.2*U*_eq_(C) or 1.5*U*_eq_ (C) for methyl H atoms.

### Crystal data

**Table:**

C_22_H_22_O_4_S·2H_2_O	*F*(000) = 444
*M**_r_* = 418.50	*D*_x_ = 1.267 Mg m^−^^3^
Orthorhombic, *A**m**m*2	Mo *K*α radiation, λ = 0.71073 Å
Hall symbol: A 2 -2	Cell parameters from 19 reflections
*a* = 19.751 (3) Å	θ = 4.9–7.6°
*b* = 10.904 (3) Å	µ = 0.18 mm^−^^1^
*c* = 5.092 (2) Å	*T* = 292 K
*V* = 1096.5 (7) Å^3^	Block, colourless
*Z* = 2	0.46 × 0.20 × 0.16 mm

### Data collection

**Table:**

Enraf–Nonius CAD-4 diffractometer	756 reflections with *I* > 2σ(*I*)
Radiation source: fine-focus sealed tube	*R*_int_ = 0.014
graphite	θ_max_ = 25.5°, θ_min_ = 1.0°
ω/2θ scans	*h* = −23→23
Absorption correction: for a sphere (*WinGX*; Farrugia, 1999)	*k* = −13→13
*T*_min_ = 0.921, *T*_max_ = 0.972	*l* = −6→3
1211 measured reflections	3 standard reflections every 50 reflections
888 independent reflections	intensity decay: 1.2%

### Refinement

**Table:**

Refinement on *F*^2^	Hydrogen site location: mixed
Least-squares matrix: full	H atoms treated by a mixture of independent and constrained refinement
*R*[*F*^2^ > 2σ(*F*^2^)] = 0.053	*w* = 1/[σ^2^(*F*_o_^2^) + (0.0747*P*)^2^ + 1.0802*P*] where *P* = (*F*_o_^2^ + 2*F*_c_^2^)/3
*wR*(*F*^2^) = 0.142	(Δ/σ)_max_ < 0.001
*S* = 1.09	Δρ_max_ = 0.52 e Å^−^^3^
888 reflections	Δρ_min_ = −0.40 e Å^−^^3^
81 parameters	Extinction correction: *SHELXL97* (Sheldrick, 2008), Fc^*^=kFc[1+0.001xFc^2^λ^3^/sin(2θ)]^-1/4^
3 restraints	Extinction coefficient: 0.011 (3)
Primary atom site location: structure-invariant direct methods	Absolute structure: Flack (1983); 284 Friedel pairs
Secondary atom site location: difference Fourier map	Flack parameter: −0.1 (2)

### Special details

**Table:**

Geometry. All e.s.d.'s (except the e.s.d. in the dihedral angle between two l.s. planes) are estimated using the full covariance matrix. The cell e.s.d.'s are taken into account individually in the estimation of e.s.d.'s in distances, angles and torsion angles; correlations between e.s.d.'s in cell parameters are only used when they are defined by crystal symmetry. An approximate (isotropic) treatment of cell e.s.d.'s is used for estimating e.s.d.'s involving l.s. planes.
Refinement. Refinement of *F*^2^ against ALL reflections. The weighted *R*-factor *wR* and goodness of fit *S* are based on *F*^2^, conventional *R*-factors *R* are based on *F*, with *F* set to zero for negative *F*^2^. The threshold expression of *F*^2^ > σ(*F*^2^) is used only for calculating *R*-factors(gt) *etc*. and is not relevant to the choice of reflections for refinement. *R*-factors based on *F*^2^ are statistically about twice as large as those based on *F*, and *R*- factors based on ALL data will be even larger.

### Fractional atomic coordinates and isotropic or equivalent isotropic displacement parameters (Å^2^)

**Table:**

	*x*	*y*	*z*	*U*_iso_*/*U*_eq_	
S1	0.5000	0.5000	0.5447 (4)	0.0400 (6)	
O1	0.5000	0.6154 (4)	0.6856 (9)	0.0545 (12)	
O2	0.0928 (2)	0.5000	−0.3050 (9)	0.0541 (12)	
H2O	0.0592 (5)	0.5000	−0.398 (4)	0.065 (8)*	
C1	0.4296 (2)	0.5000	0.3319 (11)	0.0372 (13)	
C2	0.40288 (19)	0.3902 (3)	0.2490 (10)	0.0452 (10)	
H2	0.4218	0.3167	0.3052	0.054*	
C3	0.34760 (18)	0.3895 (4)	0.0814 (10)	0.0501 (11)	
H3	0.3290	0.3156	0.0255	0.060*	
C4	0.3202 (2)	0.5000	−0.0022 (12)	0.0426 (15)	
C5	0.2615 (3)	0.5000	−0.1766 (12)	0.0445 (14)	
C6	0.2125 (2)	0.5000	−0.3109 (12)	0.0401 (13)	
C7	0.1511 (2)	0.5000	−0.4712 (13)	0.0371 (12)	
C8	0.1503 (2)	0.3873 (3)	−0.6405 (8)	0.0519 (11)	
H8A	0.1344	0.3188	−0.5396	0.078*	
H8B	0.1953	0.3708	−0.7024	0.078*	
H8C	0.1208	0.4003	−0.7877	0.078*	
O3W	0.0000 (3)	0.3082 (3)	−0.2418 (8)	0.0559 (12)*	
H1W	−0.0332	0.3467	−0.2448	0.078 (16)*	

### Atomic displacement parameters (Å^2^)

**Table:**

	*U*^11^	*U*^22^	*U*^33^	*U*^12^	*U*^13^	*U*^23^
S1	0.0347 (9)	0.0578 (12)	0.0276 (11)	0.000	0.000	0.000
O1	0.053 (2)	0.071 (3)	0.040 (3)	0.000	0.000	−0.022 (2)
O2	0.0384 (18)	0.080 (3)	0.044 (3)	0.000	0.001 (2)	0.000
C1	0.030 (2)	0.052 (3)	0.030 (3)	0.000	−0.002 (2)	0.000
C2	0.0437 (18)	0.044 (2)	0.048 (3)	0.0055 (17)	−0.0050 (19)	0.003 (2)
C3	0.0418 (19)	0.055 (2)	0.053 (3)	−0.0022 (16)	−0.010 (2)	−0.010 (2)
C4	0.029 (2)	0.064 (3)	0.035 (4)	0.000	0.004 (2)	0.000
C5	0.040 (3)	0.063 (3)	0.031 (3)	0.000	0.003 (3)	0.000
C6	0.034 (3)	0.050 (3)	0.037 (3)	0.000	0.004 (3)	0.000
C7	0.037 (2)	0.045 (3)	0.029 (3)	0.000	−0.002 (3)	0.000
C8	0.066 (2)	0.049 (2)	0.041 (3)	−0.0030 (19)	−0.002 (2)	0.000 (2)

### Geometric parameters (Å, °)

**Table:**

S1—O1	1.448 (4)	C3—H3	0.9300
S1—O1^i^	1.448 (4)	C4—C3^ii^	1.388 (5)
S1—C1	1.763 (5)	C4—C5	1.461 (7)
S1—C1^i^	1.763 (5)	C5—C6	1.185 (7)
O2—C7	1.428 (7)	C6—C7	1.462 (7)
O2—H2O	0.817 (10)	C7—C8	1.501 (5)
C1—C2^ii^	1.375 (5)	C7—C8^ii^	1.501 (5)
C1—C2	1.375 (5)	C8—H8A	0.9600
C2—C3	1.386 (5)	C8—H8B	0.9600
C2—H2	0.9300	C8—H8C	0.9600
C3—C4	1.388 (5)	O3W—H1W	0.7779
			
O1—S1—O1^i^	120.6 (4)	C3^ii^—C4—C5	119.7 (3)
O1—S1—C1	107.72 (13)	C3—C4—C5	119.7 (3)
O1^i^—S1—C1	107.72 (13)	C6—C5—C4	177.8 (6)
O1—S1—C1^i^	107.72 (13)	C5—C6—C7	178.7 (6)
O1^i^—S1—C1^i^	107.72 (13)	O2—C7—C6	109.7 (5)
C1—S1—C1^i^	104.1 (4)	O2—C7—C8	109.4 (3)
C7—O2—H2O	108.2 (13)	C6—C7—C8	109.2 (3)
C2^ii^—C1—C2	121.1 (5)	O2—C7—C8^ii^	109.4 (3)
C2^ii^—C1—S1	119.4 (3)	C6—C7—C8^ii^	109.2 (3)
C2—C1—S1	119.4 (3)	C8—C7—C8^ii^	109.9 (5)
C1—C2—C3	119.8 (4)	C7—C8—H8A	109.5
C1—C2—H2	120.1	C7—C8—H8B	109.5
C3—C2—H2	120.1	H8A—C8—H8B	109.5
C2—C3—C4	119.4 (4)	C7—C8—H8C	109.5
C2—C3—H3	120.3	H8A—C8—H8C	109.5
C4—C3—H3	120.3	H8B—C8—H8C	109.5
C3^ii^—C4—C3	120.5 (5)		
			
O1—S1—C1—C2^ii^	24.6 (5)	C2—C3—C4—C3^ii^	−0.2 (9)
O1^i^—S1—C1—C2^ii^	156.2 (4)	C2—C3—C4—C5	−179.3 (5)
C1^i^—S1—C1—C2^ii^	−89.6 (4)	C3^ii^—C4—C5—C6	−89.5 (5)
O1—S1—C1—C2	−156.2 (4)	C3—C4—C5—C6	89.5 (5)
O1^i^—S1—C1—C2	−24.6 (5)	C4—C5—C6—C7	0.00 (3)
C1^i^—S1—C1—C2	89.6 (4)	C5—C6—C7—O2	0.00 (2)
C2^ii^—C1—C2—C3	−0.7 (8)	C5—C6—C7—C8	−119.9 (3)
S1—C1—C2—C3	−179.9 (4)	C5—C6—C7—C8^ii^	119.9 (3)
C1—C2—C3—C4	0.5 (7)		

Symmetry codes: (i) −*x*+1, −*y*+1, *z*; (ii) *x*, −*y*+1, *z*.

### Hydrogen-bond geometry (Å, °)

**Table:**

*D*—H···*A*	*D*—H	H···*A*	*D*···*A*	*D*—H···*A*
O3W—H1W···O2^iii^	0.78	2.07	2.800 (6)	157

Symmetry codes: (iii) −*x*, −*y*+1, *z*.
